# Team teachers' lesson‐specific emotions and perceived instructional quality—Results of a diary study

**DOI:** 10.1111/bjep.12782

**Published:** 2025-05-03

**Authors:** Gerda Hagenauer, Franziska Muehlbacher, Christoph Helm, Christoph Weber

**Affiliations:** ^1^ Department of Educational Science and School of Education Paris Lodron University Salzburg Salzburg Austria; ^2^ Department of Educational Science and Linz School of Education Johannes Kepler University of Linz Linz Austria; ^3^ Department of Educational Science University of Education Upper Austria Linz Austria; ^4^ Research Institute for Developmental Medicine Johannes Kepler University Linz Linz Austria

**Keywords:** diary, emotions, instructional quality, spillover effects, team teachers, team teaching

## Abstract

**Background:**

Team teaching is a common practice in many schools. However, there is a lack of empirical evidence on how teachers' positive and negative affect triggered by their team‐teaching partners is related to the (perceived) instructional quality.

**Aim:**

Therefore, our goal was to investigate how positive and negative affect correlates with relevant indicators of instructional quality at a situational (i.e., lesson‐specific) level and whether spillover effects can be observed from one lesson to the next.

**Sample:**

Forty‐seven Austrian teachers participated in this longitudinal study, producing a total of 652 diary entries.

**Methods:**

The teachers wrote diary entries after each team‐teaching lesson. They rated their positive and negative affect, as well as four indicators of instructional quality (i.e., time management, clarity, differentiation and teacher–student relationship).

**Results:**

Clear links between teachers' affect and the perceived instructional quality were found at the within‐person level. The associations were less strong at the between‐person level. Spillover effects were also found, confirming the reciprocity of affect and teaching behavior.

**Conclusions:**

Emotions are evoked not only by students but also by team‐teaching partners. It is therefore important to provide teachers who engage in team teaching with training on socioemotional skills (e.g., conflict management, emotional communication).

## INTRODUCTION

Keller et al. (2014) observed that ‘for a long time, the examination of emotions was absent from academic research […]. The dearth of research on emotions is even more evident in research focused on teachers’ (p. 69). However, over the last 10 years, researchers have increasingly studied teacher emotions.

One important research topic concerns the association between teachers' emotions and instructional quality. Empirical results suggest that positive (i.e., pleasant) teacher emotions are generally associated with high instructional quality, while negative teacher emotions are not (Frenzel, 2014). This relationship, in addition to the association between emotions and teachers' (occupational) well‐being (see Burić et al., 2021; Burić & Frenzel, 2019), justifies continued research into teachers' emotions. Frenzel et al. (2015) postulated that teacher emotions are context‐specific; they vary based on the subject, class and content.

Previous studies have mainly focussed on teachers who teach alone in the classroom. However, there are many educational settings where cooperation or collaboration between teachers is required (Hargreaves & O'Connor, 2017; Vangrieken et al., 2015). For example, in team teaching, at least two teachers co‐plan, co‐instruct and co‐evaluate the same group of students in the same subject (Baeten & Simons, 2014). Team teaching is characterized by social complexity, as both the students and teaching partner may trigger emotions during interactions. Previous studies have mostly investigated students as the source of teachers' emotions in the classroom, while empirical evidence related to team‐teaching partners remains limited. One qualitative study recently showed that team teaching evokes various positive and negative emotions (Muehlbacher & Hagenauer, 2023), suggesting that it is an emotionally charged endeavour, too.

The present study focuses on the specific context of team teaching, focusing on how one teacher can trigger another teacher's emotions and how these emotions are linked to instructional quality. More concretely, we were interested in the relationship between positive and negative affect and teacher perceived instructional quality within a specific lesson (RQ1), as well as the spillover effects of positive and negative affect on perceived instructional quality across team‐taught lessons (RQ2).

### Theoretical background and conceptual framework

Researchers often understand emotions as complex phenomena comprising affective, cognitive, motivational, expressive and physiological components (Scherer & Moors, 2019; Sutton & Wheatley, 2003). For example, when a teacher experiences enjoyment, they may feel this emotion (affective), evaluate the situation positively (cognitive), feel inspired and committed (motivational), smile (expressive) and experience a quickened pulse (physiological). Furthermore, there is a distinction between studies focusing on distinct emotions, such as anger, anxiety or enjoyment (Frenzel et al., 2016) and studies organized around affect, which more generally summarize different emotions of the same valence (i.e., positive or negative) (Stark et al., 2023). While studies that refer to discrete emotions typically use categorical emotion theories, studies that refer to positive and negative affect usually rely on dimensional emotion theories. In this study, we follow the two‐dimensional model of affect (Watson & Tellegen, 1985), which describes emotional experiences in terms of its positivity (= positive affect) and negativity (= negative affect). Emotions can be classified according to their pleasantness/unpleasantness (positive, negative) and their arousal (deactivating, activating) on these two affect scales (Watson et al., 1988). Based on this distinction, a negatively valenced emotion would therefore be anger, for example. By comparison, negative affect is composed of various emotions of negative valence, such as ‘anger, contempt, disgust, guilt, fear, and nervousness’ (Watson et al., 1988, p. 1063). The difference between emotion and affect is therefore that a distinct emotion always has a very specific quality (e.g., I feel angry; I feel anxious; I feel ashamed etc.; Scherer, 2005), while positive and negative affect represents the aggregate of different negative and positive emotions that are experienced in a particular situation or event. Shuman and Scherer (2014) summarize as follows: ‘(…) it is possible to project discrete emotions onto a dimensional model (…). Indeed, it is quite common to assess discrete emotions that are then aggregated according to superordinate dimensions, factors, or clusters’ (p. 27). Applied to the present study, the aggregation of positive and negative emotions results in positive and negative affect that arise when interacting with the team‐partner in team‐teaching.

When examining emotions, it is also important to distinguish emotions from mood. Emotions typically arise from a concrete source/stimulus and are rather intense but do not last long, while moods may not have a particular source, are less intense and typically last longer (Keltner et al., 2014; Scherer, 2005; Schnall, 2010).

Another relevant distinction is between habitual (trait) and situational (state) emotions. Habitual emotions describe how teachers typically experience an emotion in a certain context (e.g., subject, class), while state emotions describe the emotional experience in a specific situation (Frenzel, 2014; Hagenauer & Hascher, 2022). The distinction between trait and state emotions is not clear‐cut (Frenzel et al., 2021). In this study, we will measure team teachers' lesson‐specific (daily) emotions (as summarized in positive and negative affect), which can be categorized as a state measure. Nevertheless, this daily state measure is less specific than measuring emotions directly in the situation as typically done when applying the experience sampling method (e.g., Becker et al., 2014, 2015; Keller & Becker, 2021).

In their conceptual model of teachers' emotions, instructional behaviour and student outcomes, Frenzel et al. (2021; see also Frenzel, 2014; Frenzel et al., 2009) adopt appraisal theory. According to appraisal theory (Scherer, 2001), the subjective cognitive evaluation of a situation elicits distinct emotions. Because people have different cognitive evaluations of the same situation, they experience different emotions. Frenzel (2014) assumes that teachers evaluate their environment based on their goals for the lesson and that different emotions arise depending on this cognitive evaluation process. Another link postulated in this conceptual model is important for the present study: emotions impact teaching behaviour and thus influence instructional quality. According to Wagner et al. (2016), instructional quality ‘comprises all teacher‐student interactions that stimulate students' cognitive, affective, and motivational development’ (p. 705). The link between emotions and instructional quality is justified by the fact that emotions influence both behaviour and cognition. For example, positive emotions typically go hand in hand with approach behaviours, while negative emotions are associated with avoidance behaviour. Negative emotions also tend to favour a rigid thinking style, while positive emotions a creative, holistic thinking style (e.g., the broaden‐and‐build theory; see Fredrickson, 2001). Consequently, it is plausible that emotions are linked to instructional quality and students' learning (Frenzel et al., 2021). Another important postulate in Frenzel et al.'s (2021) model is the reciprocity, where teachers' emotions impact instructional quality, but instructional quality also influences emotions. For example, if a teacher conducts cognitively activating lessons, they may experience pride because they are performing to a high standard and the students are engaged. The observation that the lesson is going well therefore affects the teacher's emotions. However, these reciprocal relations have not yet been tested, and most studies have assumed the first ‘direction’ of effect, where emotions affect teaching behaviour.

### Teachers' emotions and instructional quality

Research on solo teachers' emotions has provided important information on the link between emotions and instructional quality. First, previous empirical studies have generally revealed that positive emotions are associated with higher instructional quality and negative emotions are associated with lower instructional quality. However, the type of emotion, its intensity and the indicator of instructional quality can determine the nature of the associations, and particularly, the strength of the correlation. For example, Chen (2019) showed that the emotions of anger, fear and sadness were more likely to be associated with a teaching style focused on knowledge transfer, whereas the emotions of joy and love were more likely to be associated with a student‐centred approach (for similar results, see also Kalchgruber et al., 2021).

When focusing on specific aspects of instructional quality, empirical findings generally align with expectations. On a situational level, Becker et al. (2014) found a weak to moderate positive relationship between teacher enjoyment and instructional quality and weak negative relationships between anxiety and anger and instructional quality, as perceived by students. Similarly, Frenzel et al. (2009) identified positive associations between teacher enjoyment and factors like elaboration, comprehensibility, autonomy support, enthusiasm and support after failure, while anxiety and anger showed a negative relationship. In another study, Frenzel et al. (2016) confirmed positive relationships between teachers' trait enjoyment and clarity of instruction, variety in instruction, acceptance of errors, caring and support after failure, as perceived by students, while enjoyment was negatively associated with fast‐paced instruction and disrespect. These factors of instructional quality were also negatively associated with anger. Their relationships with anxiety were less clear; only acceptance of errors exhibited a significant negative correlation with teacher anxiety. Furthermore, Kalchgruber et al. (2021) demonstrated a positive relationship between positive affect and utilizing diverse teaching methods, though this relationship was non‐significant (albeit in the expected direction). Regarding teacher enthusiasm, a broader concept which encompasses teacher enjoyment (for a definition, see Keller et al., 2016), Kunter et al. (2008) showed it was positively associated with cognitive activation, monitoring and social support, as perceived by students. Similarly, Lazarides and Schiefele (2024) confirmed a positive link between teacher enthusiasm and instructional quality, particularly emotional support, as perceived by students.

Another important facet of instructional quality is the quality of teacher–student relationships, one of the core indicators for the generic dimension of ‘student support’ (Praetorius et al., 2018). Previous studies showed that teacher emotions are highly dependent on the quality of teacher–student relationships. In these studies, relationship quality was mostly understood as an antecedent of teacher emotions (Hagenauer et al., 2015; Taxer et al., 2019). However, it is possible to assume an impact in the opposite direction, as emotions and their communicative function naturally play a role in the quality of relationships (Parkinson, 1996). Thus, one can expect that the quality of teacher–student relationships and teacher emotions are mutually linked.

In sum, initial empirical findings mainly confirmed the expected association between teacher emotions and various indicators of instructional quality. However, the way this association may manifest in a team‐teaching context—with its increased focus on and promotion of teacher collaboration—has not yet been sufficiently researched.

### Team teaching—The Austrian context

Teacher cooperation takes many different forms, including team teaching, where teachers share the full responsibility for a class of students. Numerous advantages are attributed to teacher cooperation, including support for teacher professionalization, increased focus on students' needs and emotional relief. However, difficulties can also arise in teacher cooperation, including conflicts or competition among teachers (Krammer et al., 2018; Vangrieken et al., 2015).

In Austria, team‐teaching is implemented in middle schools (grades 5–8). Two teachers teach a class in mathematics, English and German. The aim of team teaching is to provide students with the best possible support by allowing, for example, internal differentiation in lessons, where students may receive different tasks, support and feedback depending on their achievement level or their motivation. Hence, team teaching aims to provide heterogeneous learner groups with individualized instruction (Baeten & Simons, 2014).

A qualitative interview study by Muehlbacher and Hagenauer (2023) showed that Austrian team teachers experience many positive and negative emotions when interacting with their team‐teaching partner. As in single‐teacher classrooms, joy and anger were frequently identified emotions in team‐taught classrooms. However, additional emotions came into play in team‐taught lessons that are typically less prominent in single‐teacher classrooms, such as gratitude, amusement or boredom. Based on Frenzel's (2014) model of teacher emotions, it is assumed that the emotions triggered by the teaching partner are linked to instructional quality. It can be assumed that positive emotions triggered by the team partner promote an approach behaviour and thus cooperation is practiced more closely. The improved cooperation should then also improve instructional quality. The opposite effect could be expected for negative emotions. These effects are also assumed if there are several days between team‐teaching lessons, as teachers are expected to prepare their subsequent lessons together between lessons. If positive emotions are present, it can be expected that joint preparation will also be more intensive compared to negative emotions.

### The present study

This study aims to contribute to the literature on teacher emotions by focusing on team teaching. We aim to investigate the link between team teachers' positive and negative affect and indicators of instructional quality. Our research is focused on the situational level, as previous studies have clearly revealed that teachers' emotions (e.g., Frenzel et al., 2015) and affect (Stark et al., 2023) vary significantly between situations. This requires an approach that accounts for intra‐individual variations in affect. By conducting a longitudinal diary study, we were able to map intra‐individual variations in teacher affect and reveal between‐teacher effects within a team‐taught lesson (Research Question 1). Furthermore, we were able to test for spillover effects and see whether teachers' positive and negative affect remained reciprocally linked to instructional quality from one lesson to the next (Research Question 2).

In German‐speaking countries, instructional quality is frequently evaluated by three generic dimensions (Alp Christ et al., 2024; Praetorius et al., 2018): classroom management, cognitive activation and student support. These dimensions are related to the indicators we selected. In classroom management, it is important to facilitate ‘time‐on‐task’ (Carroll, 1963), which refers to active learning time for students. We therefore included the indicator *time management* in the study. Cognitive activation means encouraging students to think in depth about instructional topics. The indicators we chose here were *clarity of instruction* (necessary for sparking in‐depth cognitive processes) and *differentiation*, a particularly relevant feature for team teaching (Baeten & Simons, 2014) where tasks are aligned to the individual characteristics of the learner. Finally, we focused on the quality of the *teacher–student relationship* as a core dimension of student support (Praetorius et al., 2014).

Regarding RQ1, we proposed two hypotheses:

Hypothesis 1 Positive affect triggered by the teaching partner in a team‐teaching lesson will be linked to higher teacher perceived instructional quality in the same lesson. This relationship is expected to hold at the within‐person (a) and between‐person levels (b).

Hypothesis 2 Negative affect triggered by the teaching partner in a team‐teaching lesson will be linked to lower teacher perceived instructional quality in the same lesson. This relationship is expected to hold at the within‐person (a) and between‐person levels (b).

Regarding RQ2 (spillover effects), the following hypothesis was proposed:

Hypothesis 3 Teacher perceived instructional quality in a team‐teaching lesson will have a positive effect on positive affect triggered by the teaching partner (a) and a negative effect on negative affect triggered by the teaching partner in the *next* team‐teaching lesson (b). Conversely, positive affect triggered by the teaching partner will have a positive effect on instructional quality in the *next* team‐teaching lesson (c), while negative affect triggered by the teaching partner will have a negative effect on teacher perceived instructional quality in the *next* team‐teaching lesson (d).

## MATERIALS AND METHODS

### Sample

Participants were required to be trained subject teachers currently participating in team teaching with at least one semester of previous team‐teaching experience. Forty‐seven teachers (34 female, 13 male; 14 teams, 19 teachers without their partners) participated in the diary study. They were between 22 and 62 years old (*M* = 40.5; SD = 13.3), had taught for an average of 14.1 years (SD = 13.4) and team‐taught English (*n* = 18), German (*n* = 16) and mathematics (*n* = 13).

### Data collection

The study encompassed a prequestionnaire, 15 daily diary questionnaires and a post‐questionnaire. These methods were piloted with eight team teachers. To evaluate our hypotheses, we focused on the teachers' diary entries. Throughout the study, participants were asked to focus on one team‐teaching partner in the diary with whom they taught a particular class and subject. Because affect is context‐specific, this focus ensured that the context (e.g., team partner, class and subject) remained constant.

After completing the prequestionnaire, the teachers received email links to the diary the morning of each team‐teaching lesson, so they filled in the diaries only on days when they had a team‐teaching lesson with a particular team‐teaching partner and predefined class of students. They were asked to fill out this diary shortly after their team‐teaching lesson. At the latest, the entries had to be completed by midnight of the same day. The diary questionnaires ended after 15 entries. Data collection for the diary entries lasted from October 2022 to March 2023 because participants started at different times, they had different amounts of team‐teaching lessons per week, and certain events sometimes prevented them from filling in the diary entries (e.g., illness, tests that encompassed the entire lesson or out‐of‐school activities such as skiing weeks). Overall, the teachers filled in 652 diary entries, with a 92% completion rate. The attrition rate was as follows: for measurement occasion 1 to 4, all entries were available. For the fifth occasion, requested values for one teacher were missing. Numbers of missing data for the other occasions 6 (O6) to 15 (O15) were 2 (O6), 3 (O7), 4 (O8‐O10), 6 (O11‐O12), 7 (O13), 8 (O14) and 9 (O15). Eight further teachers reported some but not all requested information. For treatment of missing data, see the data analysis section.

### Measures

#### Positive and negative affect

For the daily questionnaires, teachers were asked to rate the positive and negative affect in the preceding team‐teaching lesson due to their partner. The teachers were asked to finish the sentence: ‘Today during the lesson in the selected class my team‐teaching partner made me feel… [active, attentive, interested, excited, alert, grateful, determined, enthusiastic, inspired, amused, proud, distressed, jittery, upset, irritable, bored, nervous, guilty, scared, hostile, ashamed, afraid]’. They were provided with a list of 11 positive and 11 negative emotions, based on the German version of the Positive and Negative Affect Schedule (Breyer & Bluemke, 2016; PANAS) and Muehlbacher and Hagenauer's (2023) study. In this study, Muehlbacher and Hagenauer (2023) found that ‘gratitude’, ‘amusement’ and ‘boredom’ are particularly relevant in the team‐teaching setting, which is why they were added to the diaries, upon being approved by the teams that piloted the diary study. The emotion ‘strong’, usually included in the PANAS, was deleted, as suggested by the piloting teams. Participants then rated their intensity on a scale of 1 (*Not at all*) to 5 (*Extremely*). For each diary entry, total scores were calculated for both positive and negative affects by averaging each teacher's responses on the provided list of positive and negative emotions. The reliability of both scales is satisfactory. Omega (estimated based on a multilevel model) is .89 for positive affect at within level and .92 at between level. Omega for negative affect is .73 (within) and .92 (between). Additionally, we calculated Omega separately for each of the 15 measurement occasions. Values range from .94 to .97 for positive affect and from .82 to .96 for negative affect.

#### Indicators of instructional quality

The teachers provided information on the following indicators: *time management* (Kunter et al., 2017; ‘I often had the impression that a lot of time was wasted in my lesson’; recoded), *clarity of instruction* (Frey et al., 2009; ‘The essential things were clear to the students’), *differentiation* (Mang et al., 2023; ‘The students received individual support if they had difficulty understanding a topic or an assignment’) and the *teacher–student relationship* (Aldrup et al., 2018; ‘The students respected me’). Finally, on a scale of 1 (*Does not apply*) to 4 (*Fully applies*), the teachers were asked to rate the extent to which these statements applied to the respective team‐teaching lesson.

To encourage teachers' participation over several weeks, we opted for single‐item measures to assess instructional quality. We selected the item that we felt best and most broadly represented the essence of each construct. In other words, this choice was based on a content validity check of the respective item. As only one item was recorded for each dimension of instructional quality, we cannot report a specific reliability measure. However, the correlations between the indicators indicate (see Table 1) that they measure distinct aspects of instructional quality. Since the correlations are not only in the expected direction across the four indicators of instructional quality (convergent validity) but also with positive and negative affect (concurrent validity), they provide initial support for the construct validity of the indicators (for a critical discussion on the reliability and validity of single item measures, see Allen et al., 2022).

**TABLE 1:** Descriptive statistics and intercorrelations.

	PA	NA	TM	CI	DI	TS	*M* (between)	SD_b_	SDw
PA	‐	−.204**	.170**	.248**	.145**	.152*	3.547	.881	.465
NA	−.191	‐	−.120	−.267**	−.052	−.176**	1.095	.235	.173
TM	.129	−.212*	‐	.168**	.111	.107	3.562	.706	.644
CI	.413**	−.174	.392*	‐	.173**	.237**	3.551	.617	.531
DI	.508**	−.351*	.389*	.514**	‐	.128**	3.528	.634	.532
TS	.166	−.158	.431*	.357*	.253	‐	3.810	.412	.290
ICC	.71	.45	.17	.28	.30	.50			

*Note*: Estimates above the diagonal refer to intercorrelations on the within‐person level; estimates below the diagonal refer to the between‐person level. *N* = 47 (between) and *N* = 653 (within).

Abbreviations: CI, clarity of instruction; DI, differentiation; ICC, intra‐class correlation; NA, negative affect; PA, positive affect; TM, time management; TS, teacher–student relationship.

* *p* < .05.

** *p* < .01.

### Data analysis

R version 4.3.1 (R Core Team, 2023) and the packages haven (Wickham et al., 2023), psych (Revelle, 2023) and nlme (Pinheiro et al., 2023) were used. We also utilized the MplusAutomation package (Hallquist & Wiley, 2018) to connect R and Mplus.

Dynamic structural equation modelling was applied in Mplus version 8 (Muthén & Muthén, 1998‐2017). Dynamic structural modelling combines time‐series modelling, multilevel modelling and structural equation modelling.

The time interval between two consecutive measurement occasions mainly varied between 1 day and 14 days, although some intervals were longer due to holidays. To account for these unequal time intervals, we computed the time variable in day units, based on the timestamps of data collection (where a lag of 1 represents a 1‐day difference)^1^ and applied the TINTERVAL option in M*plus*. This approach converted the unequal‐interval problem to a missing‐data problem by adding missing data for days with no realized measurement. We set the time interval value to 1, preserving the day‐based time scale, so that lagged effects refer to effects of a variable (within‐person deviation from the individual mean) at day *t‐1* on a variable (within‐person deviation from the individual mean) at day *t*. This procedure is effective with up to 80%–90% of missing data (Hamaker et al., 2023; McNeish & Hamaker, 2020). In the current study, the rate of missing data was slightly below 80% (79.2%–79.4%).

**FIGURE 1:**
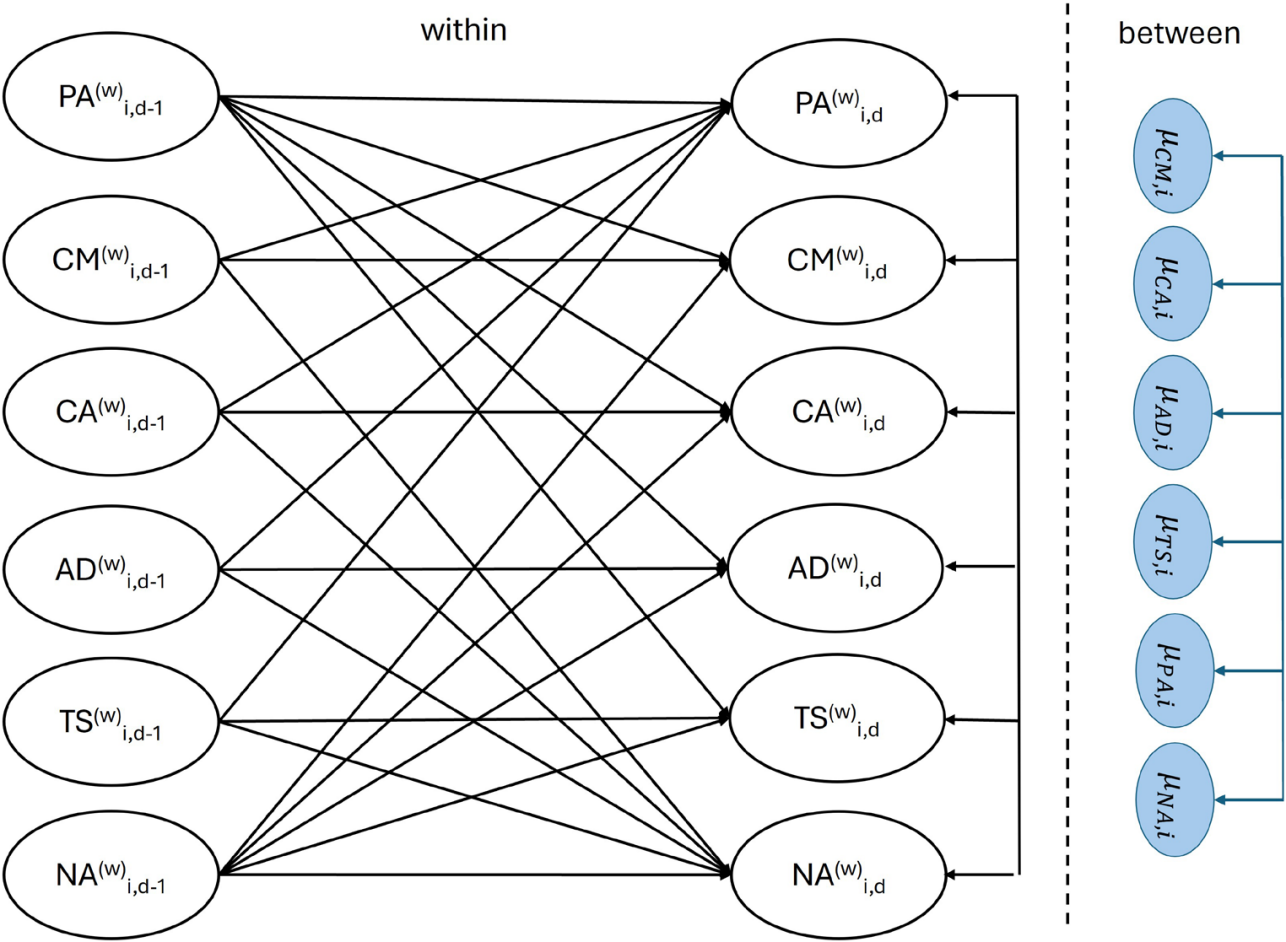
A graphical representation of the DSEM Model. Observed variables were decomposed into latent within‐ and between‐person components (left part of the figure). Auto‐ and cross‐regressive effects were estimated on the within‐person level. Correlations among the random intercepts were estimated on the between‐person level (right part of the figure).

Figure 1 shows a schematic illustration of the model. At the within‐person level, we included autoregressive effects for all six variables, where, for example, instructional quality on day *t* was regressed on the same measure of instructional quality assessed at day *t‐1*. The same was done for positive and negative affect. Additionally, the four variables related to instructional quality at day *t‐1* were included as lagged predictors of both positive and negative affect assessed on the following day *t* (spillover effects). Positive and negative affect at day *t‐1* were, in turn, added as lagged predictors of instructional quality on the next day *t* (reverse spillover effects).

Moreover, we estimated the residual covariances between the variables that were assessed simultaneously. For H1 and H2, we assumed an average positive correlation between any two variables assessed on the same day.

Missing values were treated by Full Information Bayesian (FIB) Estimation, which is akin to Full Information Maximum Likelihood (FIML) but adapted for the Bayesian framework. Hence, missing values were assumed to be random given all variables in the model. Bayesian estimators are required for DSEM. We maintained the Mplus settings of 50% burn‐in duration and diffuse priors for the current analysis. Based on two Markov chain Monte Carlo (MCMC) chains with 3000 iterations, the results were only retained after the 50th iteration (thinning = 50) to minimize autocorrelation between the MCMC draws (see Asparouhov et al., 2018; Hamaker et al., 2018; McNeish & Hamaker, 2020).

The estimates presented here are the 95% credible intervals, corresponding to the medians of the derived posterior parameter distributions. The parameters that did not contain zero in their 95% credible interval were deemed to be substantially different from zero. This is comparable to the two‐tailed 5% α‐level utilized in all other analyses presented in this work.

## RESULTS

### Descriptive statistics and intercorrelations

As shown in Table 1, the daily instructional quality dimensions and affect were partially correlated. At the within‐person level, positive affect was positively related to all dimensions of instructional quality. However, on the between‐person level, positive affect was only positively related to clarity of instruction and differentiation. Moreover, negative affect had a significant, negative relationship with clarity of instruction and teacher–student relationship at the within‐person level and with effective time management and differentiation on the between‐person level.

Intra‐class correlation values point to substantial variances at the between‐person level as well as the within‐person level. In comparison, the variance at the within‐person level was higher for negative affect (55%; ICC = .45) compared to positive affect (29%; ICC = .71), suggesting that negative affect more strongly varied within a teacher across situations. In terms of instructional quality, the perceived quality of the teacher–student relationship was equally influenced by situational as well as contextual factors (ICC = .50), while the other dimensions of instructional quality varied more within teachers across situations (70%–83%; ICCs from .17 to .30) than between teachers.

### Research question 1: Relationship between affect and instructional quality

The two chains of the DSEM converged. The potential reduction of the scale was 1.003. The variance explained ranged between 2.6% and 9.6%, except for negative affect, for which the model explained a higher share of variance (19.3%).

Concerning Hypotheses 1 and 2, our findings (see Table 2) show that at the within‐person level, the correlations between the variables examined were largely moderate and statistically significant. Positive affect was negatively associated with negative affect (*r* = −.227 [−.315, −.136]) and positively correlated with the measures of instructional quality (ranging from *r* = .146 [.062, .232] for to *r* = .240 [.158, .324]). Negative affect was correlated with clarity of instruction (*r* = −.262 [−.352, −.173]) and teacher–student relationship (*r* = −.190 [−.282, −.099]), but not with time management (*r* = −.066 [−.165, .031]) and differentiation (*r* = .007 [−.090, .102]). Hence, H1a and H2a are (mostly) confirmed by the data. Moreover, the four instructional quality variables were positively associated with correlations ranging from *r* = .096 [.010, .188] to *r* = .230 [.146, .306].

**TABLE 2:** Results from the dynamic structural equation model: reciprocal relations of daily instructional quality dimensions and positive and negative affect.

		Unstandardised results			Standardized results		
		Estimate	Lower 2.5% CI	Upper 2.5% CI	Estimate	Lower 2.5 % CI	Upper 2.5% CI
*Spillover effects*							
PA ON	TM&1	.**117**	.**017**	.**205**	.**162**	.**024**	.**286**
PA ON	CI&1	−.012	−.132	.116	−.014	−.149	.134
PA ON	DI&1	.051	−.066	.165	.058	−.076	.192
PA ON	TS&1	−.019	−.253	.219	−.012	−.157	.136
NA ON	TM&1	.003	−.025	.048	.011	−.096	.121
NA ON	CI&1	.**045**	.**002**	.**082**	.**137**	.**006**	.**254**
NA ON	DI&1	−.031	−.066	.007	−.095	−.202	.022
NA ON	TS&1	−.037	−.114	.039	−.063	−.193	.065
TM ON	PA&1	.155	−.306	.367	.111	−.225	.265
TM ON	NA&1	**−.530**	**−.997**	**−.082**	**−.143**	**−.264**	**−.022**
CI ON	PA&1	.097	−.050	.229	.085	−.044	.201
CI ON	NA&1	−.190	−.543	.175	−.062	−.177	.056
DI ON	PA&1	−.015	−.155	.157	−.013	−.134	.138
DI ON	NA&1	**−.350**	**−.674**	**−.002**	**−.114**	**−.219**	**−.001**
TS ON	PA&1	.056	−.033	.136	.090	−.055	.221
TS ON	NA&1	−.008	−.211	.200	−.005	−.126	.118
*Carry over effects*							
PA ON	PA&1	.122	−.024	.258	.122	−.024	.258
NA ON	NA&1	.**428**	.**322**	.**526**	.**428**	.**322**	.**526**
TM ON	TM&1	−.039	−.198	.140	−.039	−.198	.140
CI ON	CI&1	.003	−.124	.140	.003	−.124	.140
DI ON	DI&1	.115	−.011	.242	.115	−.011	.242
TS ON	TS&1	.077	−.100	.211	.077	−.110	.211
*Within‐level covariance/correlation*							
PA WITH	NA	**−.016**	**−.023**	**−.009**	**−.227**	**−.315**	**−.136**
PA WITH	TM	.**051**	.**024**	.**082**	.**180**	.**087**	.**277**
PA WITH	CI	.**058**	.**037**	.**081**	.**240**	.**158**	.**324**
PA WITH	DI	.**036**	.**015**	.**059**	.**152**	.**064**	.**240**
PA WITH	TS	.**019**	.**008**	.**031**	.**146**	.**062**	.**232**
NA WITH	TM	−.006	−.016	.003	−.066	−.165	.031
NA WITH	CI	**−.022**	**−.030**	**−.014**	**−.262**	**−.352**	**−.173**
NA WITH	DI	.001	−.007	.008	.007	−.090	.102
NA WITH	TS	**−.008**	**−.013**	**−.004**	**−.190**	**−.282**	**−.099**
TM WITH	CI	.**051**	.**021**	.**083**	.**152**	.**062**	.**239**
TM WITH	DI	.**036**	.**006**	.**066**	.**106**	.**016**	.**194**
TM WITH	TS	.**017**	.**002**	.**035**	.**096**	.**010**	.**188**
CI WITH	DI	.**047**	.**023**	.**071**	.**169**	.**085**	.**247**
CI WITH	TS	.**035**	.**022**	.**049**	.**230**	.**146**	.**306**
DI WITH	TS	.**021**	.**008**	.**034**	.**135**	.**052**	.**215**
*Between‐level covariance/correlations*							
PA WITH	NA	−.027	−.101	.028	−.180	−.502	.168
PA WITH	TM	.035	−.089	.178	.117	−.268	.466
PA WITH	CI	.**138**	.**021**	.**317**	.**413**	.**065**	.**667**
PA WITH	DI	.**177**	.**056**	.**374**	.**503**	.**181**	.**722**
PA WITH	TS	.049	−.061	.186	.169	−.187	.484
NA WITH	TM	−.010	−.045	.017	−.160	−.525	.240
NA WITH	CI	−.013	−.050	.013	−.190	−.534	.185
NA WITH	DI	−.024	−.064	.003	−.331	−.632	.048
NA WITH	TS	−.010	−.040	.013	−.171	−.488	.193
TM WITH	CI	.052	−.004	.137	.372	−.029	.667
TM WITH	DI	.053	−.004	.139	.365	−.035	.664
TM WITH	TS	.**052**	.**007**	.**122**	.**427**	.**058**	.**702**
CI WITH	DI	.**080**	.**021**	.**181**	.**504**	.**149**	.**742**
CI WITH	TS	.049	.000	.122	.364	−.003	.644
DI WITH	TS	.036	−.019	.108	.255	−.120	.576

Explained variance (level‐1 *R* ^2^)	Estimate	Lower 2.5% CI	Upper 2.5% CI
POS	.**073**	.**020**	.**158**
NEG	.**205**	.**128**	.**292**
TM	.**055**	.**011**	.**127**
CI	.**022**	.**002**	.**066**
DI	.**034**	.**007**	.**092**
TS	.**026**	.**003**	.**081**

*Note*: Table displays unstandardized and standardized regression coefficients and the corresponding 95% credible interval. Effects whose 95% credible interval does not contain zero are highlighted in bold face. *N* = 47 (between) and *N* = 653 (within).

Abbreviations: CI, clarity of instruction; CI&1, clarity of instruction at the previous team‐teaching lesson; DI, differentiation; DI&1, differentiation at the previous team‐teaching lesson; NA, negative affect; NA&1, negative affect at the previous team‐teaching lesson; PA, positive affect; PA&1, positive affect at the previous team‐teaching lesson; TM, time management; TM&1, time management at the previous team‐teaching lesson; TS, teacher–student relationship; TS&1, teacher–student relationship at the previous team‐teaching lesson.

At the between‐person level (H1b and H2b), the following relationships were observed over time: First, clarity of instruction (*r* = .413 [.065, .667]) and differentiation (*r* = .503 [.181, .722]) were positively associated with positive affect. Second, none of the variables was significantly associated with negative affect. Third, the four dimensions of instructional quality were interrelated at a moderate to strong level (*r* = .255 [−.120, .576]) to (*r* = .504 [.149, .742]). However, only two correlations were statistically significant, mainly due to the low sample size at the between‐person level.

### Research question 2: Spillover effects

Spillover effects, as outlined in Hypothesis 3, were confirmed in four cases. First, time management in the team‐teaching lesson positively influenced positive affect on the next team‐teaching lesson (*β* = .162 [.024, .286]). Second, and contradicting Hypothesis 3, clarity of instruction in the team‐teaching lesson had a positive effect on negative affect on the next team‐teaching lesson (*β* = .137 [.006, .254]). Third, negative affect was negatively correlated with time management in the next team‐teaching lesson (*β* = −.143 [−.264, −.022]). Fourth, negative affect had a negative lagged effect on differentiation (*β* = −.114 [−.219, −.001]). Referring to empirically derived benchmarks for cross‐lagged effects (Orth et al., 2024), the effect sizes can be regarded as medium to large (.03 = small effects; .07 = medium effects and .12 = large effects).

A statistically significant carry over effect (autoregressive paths) was only observed for negative affect (*β* = .428 [.322, .526]).

Notably, we also conducted a set of further analyses to check the robustness of the results (bivariate analyses considering only one instructional variable and one affect variable at a time—see Table 2S in the supplement; and analyses controlling for time trends—see Table 1S in the supplement). These analyses confirm the findings reported above.

## DISCUSSION

In this study, we investigated the relationship between team teachers' positive and negative affect and perceived instructional quality in team‐teaching classrooms. We were also interested in spillover effects. To test these relationships, we relied on a sample of Austrian teachers who completed diaries to reflect on their experiences in their previous team‐teaching lessons.

First, our results showed that negative affect triggered by a teaching partner varies more within teachers across situations when compared to positive affect. This finding suggests that positive emotions may depend more on the general compatibility between teaching partners, while negative emotions seem to be influenced more by the specific dynamics of teamwork in the classroom. However, compared to solo teaching, the variability in teachers' affect within the team‐teaching context is generally lower. Frenzel et al. (2015), for example, found a within‐teacher variability of 83% for enjoyment, 80% for anger and 74% for anxiety (see also Becker et al., 2015). This difference could be due to the fact that positive and negative affect were measured in this study rather than distinct emotions, as in Frenzel et al.'s (2015) study. It may also be explained by the fact that, in the previous studies, teacher emotions were largely driven by interactions with many different students in class. In this study, the focus was only on one person—namely the teaching partner. Furthermore, this difference could also be due to the fact that we did not assess positive and negative affect in the specific situation, but the affective experience referred to the entire lesson. This could contribute to less variation. In addition, the teachers had time to complete the diary until midnight. This could also have led to the trait portion of positive and negative affect playing a greater role in the retrospective responses.

In this context, we would also like to briefly point out that carry over effects were only found for negative affect but not for positive affect in the DSEM (comment: In the bivariate models the carry over effect for positive affect just reached significance). The finding could be interpreted to suggest that negative emotions are more deeply ingrained in memory than positive emotions (Baumeister et al., 2001). This means that it is more likely that teachers who have experienced negative affect in one lesson will still be feeling it in the next.

Regarding teaching quality indicators, the quality of the teacher–student relationship is significantly less influenced by situational factors than the other indicators. This finding seems plausible, since established social relationships tend not to fluctuate significantly from lesson to lesson. However, the high variation in other teaching quality indicators within individual teachers is noteworthy. By comparison, Praetorius et al. (2014) found more stable values for solo teachers' instructional quality (based on expert assessments). One interpretation is that co‐teaching may lead to more frequent variations in teaching quality. Additionally, teachers' different expectations of what constitutes high‐quality teaching could play a role. For example, teachers may rate their co‐teacher's instruction less favourably if their expectations for teaching quality differ.

Moving on to Hypotheses 1a and 2a, our results showed that there were significant associations between teachers' positive and negative affect and perceived instructional quality at the within‐person level (see also Becker et al., 2014, Frenzel et al., 2016; Kalchgruber et al., 2021 for solo teachers). Specifically, teachers who experienced positive affect during a lesson reported more effective time management, greater clarity of instruction, greater differentiation and stronger teacher–student relationships in the same lesson. These relationships were negative for negative affect, except for differentiation and time management, which had no significant association with negative affect. On the between‐person level (H1b and H2b), only two associations were significant: Teachers who reported positive affect also reported greater clarity of instruction and differentiation.

When comparing the strength of correlations between positive and negative affect and instructional quality in the same lesson, both within‐ and between‐teachers, the relationship with clarity of instruction was the strongest and most consistent across all four correlations. This robust relationship might be explained by the fact that the item assessing clarity of instruction focused on whether teachers felt they effectively conveyed key content to their students. Teachers may have interpreted this as a sign of their teaching effectiveness. When teachers responded affirmatively to this item, they may have felt they successfully supported their students' learning process, which is a core objective for teachers and closely linked to teachers' emotions (see Frenzel's model on teacher emotions, 2014).

A somewhat counterintuitive finding emerged when examining the spillover effects between negative affect and clarity of instruction (Hypothesis 3). Contrary to our expectations, we found that a high level of perceived clarity in one team‐taught lesson was associated with higher negative affect in the next lesson. It is rather difficult to interpret this result in terms of content. One initial hypothesis could be that teachers who were very satisfied with the clarity of the instruction in a lesson (i.e., the students understood the content very well) might have very high expectations of the next lesson. If the team partner was unable to meet these high expectations in the team teaching and the students may have understood the subject matter less well, this could lead to higher negative affect in the interaction with the team partner. Alternatively, a lesson that is experienced as very successful could also lead to feeling overconfident (in the sense of the deactivating function of emotions), investing less time in preparation, which in turn leads to higher negative affect in the next lesson. In this context, it also needs to be mentioned that in the additional bivariate analyses (see Supplement Table 2S), this spillover effect just missed the significance threshold. However, the direction is the same as in the complete DSEM. Further research is needed to determine whether this finding holds across contexts and samples.

Other spillover effects related to Hypothesis 3 followed the expected direction. Teachers who experienced effective time management in one lesson reported higher positive affect in the following lesson. Additionally, teachers who experienced negative affect in one lesson reported less effective time management and lower differentiation in the following lesson.

These spillover effects between negative affect and both time management and differentiation are interesting because, at the within‐person level and in the same lesson, these associations were not significant. This suggests that negative affect may have a delayed effect on teaching quality in a team‐teaching context. This is especially plausible for differentiation; since teachers usually plan lessons in advance, they are likely to implement differentiation strategies even if they experience negative emotions in the lesson due to the team partner. However, negative emotions may lead teachers to be less likely to collaborate in planning differentiated instruction in the future. Negative emotions could trigger avoidance behaviour, causing differentiated instruction—which often requires strong cooperation—to suffer as a result. Similar dynamics could apply to effective time management. If teachers experience negative emotions while interacting with their teaching partner during a lesson, this may hinder their cooperation and make them appear less unified in subsequent lessons, which might negatively impact time management. These are initial interpretations that require further investigation in future studies. Overall, the findings align with previous research which has shown that teachers' emotions—particularly negative emotions like anger and anxiety—are closely related to indicators of classroom management (de Ruiter et al., 2019; Hagenauer et al., 2015).

Finally, we would like to address the few significant correlations between affect and perceived instructional quality at the between‐person level during the same lesson (Hypothesis 1b and 2b). Although non‐significant, the direction of the correlations consistently aligned with our expectations. The magnitude of the correlations was also similar to the within‐person level. It is therefore possible that some of the non‐significant correlations could be attributed to the small sample size at level 2. For this reason, we refrain from making any content‐related interpretations at this point. Future studies should aim to recruit larger samples of team teachers to better investigate these relationships.

### Study limitations and implications for future research

Although this study allowed us to examine team teachers' positive and negative affect on a situational and longitudinal level for the first time, some limitations should be considered when interpreting the results. First, the results rely on teachers' self‐reports. While self‐assessments of emotions are valuable for capturing the affective core of an emotion, they are more prone to bias in terms of instructional quality. Studies have shown that student and teacher ratings of instructional quality do not correlate strongly, with teacher ratings typically being more favourable than student ratings (Wagner et al., 2016). Hence, instructional quality may have been somewhat overestimated in this study. In future studies, it would be useful to assess instructional quality via the perception of students or experts. Second, although teachers were instructed to fill out the diaries as soon as possible after the team‐teaching lesson, after considering the busy nature of school days and after‐school commitments, we extended the deadline until midnight of the same day. As a downside of this decision, there was an increased risk of recall bias. In future studies, it would therefore be advisable to have the diary completed directly after the lesson. Third, we used single items to measure facets of instructional quality, which is common practice in studies using intensive longitudinal methods. However, Dejonckheere et al. (2022) demonstrated that single‐item measures are prone to bias, potentially exaggerating the variability of a construct across situations. We also did not systematically test the reliability and validity of the single‐item measures before the study was conducted; rather, we chose the items due to content‐related considerations. Hence, a systematic validation of single‐item measures to assess instructional quality (in team‐teaching settings) is still lacking. Single items are increasingly being used in psychological research, particularly in intensive longitudinal studies, as they have several advantages (e.g., economical use). This makes systematic validation studies of single‐item measures even more important for the future (see for example Allen et al., 2022). Fourth, even though we accounted for the time intervals between measurements, it should be noted that the time windows between some team‐teaching lessons were relatively long. Consequently, it was more difficult to observe spillover effects than it might have been if consecutive lessons were analysed. Fifth, to gain some initial insights into the team‐teaching context, we considered it useful to use positive or negative affect as a general indicator of emotional experience. However, since distinct emotions have unique associations with teaching behaviour, future studies should focus on concrete emotions. Lastly, as mentioned above, the varying intervals between diary entries could have led to an underestimation of spillover effects.

## CONCLUSION

The results of this study are significant because they show that emotions triggered by a teaching partner—not just those triggered by students—are associated with the perceived instruction quality of that lesson and, in some cases, subsequent lessons as well. We were therefore able to demonstrate some of the reciprocal effects postulated in the model of teacher emotions (Frenzel, 2014; Frenzel et al., 2021) for team‐teaching lessons. Teachers' negative affect especially appears to have a spillover effect on the next team‐teaching lesson. It is particularly worth noting that differentiation, which should be encouraged in team‐teaching lessons, appears to suffer when teachers experience negative emotions triggered by an interaction with their teaching partner during a lesson. To mitigate these negative emotions and prevent them from having long‐term negative effects on instructional quality in team‐taught lessons, it is important to promote teachers' social–emotional competence (Carstensen et al., 2019). Teacher training should focus on fostering emotionally positive cooperation and equipping teachers with the skills to navigate emotionally challenging situations effectively. Establishing shared goals and strategies for lesson design can also provide a strong foundation for positive emotional experiences in the team‐teaching classroom.

## AUTHOR CONTRIBUTIONS

**Gerda Hagenauer:** Conceptualization; writing – original draft; writing – review and editing; project administration; supervision. **Franziska Muehlbacher:** Conceptualization; writing – original draft; writing – review and editing; project administration; data curation; investigation. **Christoph Helm:** Writing – original draft; writing – review and editing; formal analysis. **Christoph Weber:** Writing – review and editing; formal analysis.

## FUNDING INFORMATION

We did not receive any financial support.

## CONFLICT OF INTEREST STATEMENT

We have no conflict of interest to disclose.

## Supporting information


Data S1:

## Data Availability

The data that support the findings of this study are available from the corresponding author upon reasonable request.

## Footnotes

^1^
The time of completion of the questionnaire was not recorded. Therefore, days are the smallest possible time interval.
