# Supplementary material for: Team teachers' lesson‐specific emotions and perceived instructional quality—Results of a diary study
Source: Br J Educ Psychol. 2025 May 3;95(Suppl 1):S194–211. doi: 10.1111/bjep.12782 (PMC12427169; doi:10.1111/bjep.12782)
Supplement: Supplementary file 1 — Data S1: [file BJEP-95-S194-s001.docx]

**Online Supplement for the Manuscript**

***Team teachers’ lesson-specific positive and negative affect and perceived instructional quality – Results of a diary study.***

**Controlling for trend in Dynamic Structural Equation Models (DSEM)**

When there is trend in the data (i.e. the means of an outcome decreases or increases over time), it may be necessary to detrend (i.e. control for trends) the data (McNeish & Hamaker, 2020). To check the robustness of the results of the used DSEM without controlling for trend, we provide here also detrended results. There are generally two approaches used to address time trends (Fang et al., 2024; McNeish & Hamaker, 2020). On the one hand, a DSEM including time as a predictor on within level could be used. On the other hand, a residual DSEM (rDSEM) enables modelling of time trends. rDSEM models lagged relations between residuals and not between the within-person components (Hamaker et al., 2023). The results of both models are shown in Table 1S. Although there were significant trends for four out of the six variables, there were only slight differences between the results of the different models. In regard to the significant effects of the DSEM (Section A), it is noticeable that the time-lagged effect of negative affect on time management was not significant in the DSEM controlling for trend (Section B). But this effect was also significant in the rDSEM controlling for trend (Section C). Therefore, both the DSEM controlling for trend and the rDSEM controlling for trend support the results of the DSEM.

**Bivariate Dynamic Structural Equation Models**

Results of bivariate DSEM models which consider only one affect variable and one instructional quality variable at a time are reported in Table 2S. The bivariate results supported the multivariate findings. The following minor discrepancies should be mentioned. First, although of similar size, the time-lagged effect of clarity of instruction on negative affect was not significant in the bivariate DSEM. However, the 95%-CI overlapped only just zero. Second, the same was found for the effects of negative affect on differentiation. Third, the autoregressive (carry over) effect of positive affect was significant in the bivariate analyses, but not in the multivariate.

**Table 1S**

*Results from the DSEM reported in the paper, a DSEM controlling for trend, and a residual DSEM (rDSEM) controlling for trend.*

|  |  | **A. DSEM** | | | | | |  | **B. DSEM controlling for trend** | | | | | |  | **C. rDSEM controlling for trend^a^** | | |
| --- | --- | --- | --- | --- | --- | --- | --- | --- | --- | --- | --- | --- | --- | --- | --- | --- | --- | --- |
|  |  | Unstandardised Results | | | Standardised Results | | |  | Unstandardised Results | | | Standardised Results | | |  | Unstandardised Results | | |
|  |  | Est. | 2.5%  CI low | 2.5%  CI up | Est. | 2.5%  CI low | 2.5%  CI up |  | Est. | 2.5%  CI low | 2.5%  CI up | Est. | 2.5%  CI low | 2.5%  CI up |  | Est. | 2.5%  CI low | 2.5%  CI up |
|  |  |  |  |  |  |  |  |  |  |  |  |  |  |  |  |  |  |  |
| spillover effects | |  |  |  |  |  |  |  |  |  |  |  |  |  |  |  |  |  |
| PA ON | TM&1 | **0.117** | **0.017** | **0.205** | **0.162** | **0.024** | **0.286** |  | **0.115** | **0.015** | **0.201** | **0.16** | **0.02** | **0.281** |  | **0.114** | **0.013** | **0.204** |
| PA ON | CI&1 | -0.012 | -0.132 | 0.116 | -0.014 | -0.149 | 0.134 |  | -0.014 | -0.132 | 0.110 | -0.014 | -0.151 | 0.127 |  | -0.004 | -0.122 | 0.115 |
| PA ON | DI&1 | 0.051 | -0.066 | 0.165 | 0.058 | -0.076 | 0.192 |  | 0.049 | 0.169 | 0.169 | 0.055 | -0.087 | 0.193 |  | 0.044 | -0.075 | 0.152 |
| PA ON | TS&1 | -0.019 | -0.253 | 0.219 | -0.012 | -0.157 | 0.136 |  | -0.030 | -0.280 | 0.201 | -0.019 | -0.178 | 0.127 |  | -0.028 | -0.225 | 0.171 |
|  |  |  |  |  |  |  |  |  |  |  |  |  |  |  |  |  |  |  |
| NA ON | TM&1 | 0.003 | -0.025 | 0.048 | 0.011 | -0.096 | 0.121 |  | 0.003 | -0.027 | 0.032 | 0.01 | -0.099 | 0.119 |  | 0.004 | -0.027 | 0.032 |
| NA ON | CI&1 | **0.045** | **0.002** | **0.082** | **0.137** | **0.006** | **0.254** |  | **0.042** | **0.000** | **0.080** | **0.131** | **0.001** | **0.248** |  | **0.041** | **0.001** | **0.075** |
| NA ON | DI&1 | -0.031 | -0.066 | 0.007 | -0.095 | -0.202 | 0.022 |  | -0.032 | -0.068 | 0.006 | -0.098 | -0.209 | 0.017 |  | -0.024 | -0.054 | 0.009 |
| NA ON | TS&1 | -0.037 | -0.114 | 0.039 | -0.063 | -0.193 | 0.065 |  | -0.045 | -0.122 | 0.038 | -0.075 | -0.206 | 0.063 |  | -0.025 | -0.083 | 0.039 |
|  |  |  |  |  |  |  |  |  |  |  |  |  |  |  |  |  |  |  |
| TM ON | PA&1 | 0.155 | -0.306 | 0.367 | 0.111 | -0.225 | 0.265 |  | 0.129 | -0.325 | 0.364 | 0.092 | -0.235 | 0.263 |  | 0.031 | -0.334 | 0.322 |
| TM ON | NA&1 | **-0.530** | **-0.997** | **-0.082** | **-0.143** | **-0.264** | **-0.022** |  | -0.453 | -0.903 | 0.010 | -0.122 | -0.244 | 0.003 |  | **-0.456** | **-0.893** | **-0.004** |
| CI ON | PA&1 | 0.097 | -0.050 | 0.229 | 0.085 | -0.044 | 0.201 |  | 0.086 | -0.066 | 0.364 | 0.075 | -0.056 | 0.2 |  | 0.075 | -0.073 | 0.219 |
| CI ON | NA&1 | -0.190 | -0.543 | 0.175 | -0.062 | -0.177 | 0.056 |  | -0.232 | -0.570 | 0.010 | -0.075 | -0.182 | 0.044 |  | -0.22 | -0.543 | 0.122 |
| DI ON | PA&1 | -0.015 | -0.155 | 0.157 | -0.013 | -0.134 | 0.138 |  | -0.016 | -0.162 | 0.176 | -0.014 | -0.142 | 0.153 |  | 0.01 | -0.145 | 0.192 |
| DI ON | NA&1 | **-0.350** | **-0.674** | **-0.002** | **-0.114** | **-0.219** | **-0.001** |  | **-0.364** | **-0.698** | **-0.013** | **-0.117** | **-0.223** | **-0.005** |  | **-0.349** | **-0.68** | **-0.025** |
| TS ON | PA&1 | 0.056 | -0.033 | 0.136 | 0.090 | -0.055 | 0.221 |  | 0.056 | -0.034 | 0.138 | 0.09 | -0.055 | 0.223 |  | 0.05 | -0.042 | 0.135 |
| TS ON | NA&1 | -0.008 | -0.211 | 0.200 | -0.005 | -0.126 | 0.118 |  | -0.016 | -0.221 | 0.171 | -0.009 | -0.132 | 0.102 |  | -0.022 | -0.218 | 0.163 |
| carry-over effects | |  |  |  |  |  |  |  |  |  |  |  |  |  |  |  |  |  |
| PA ON | PA&1 | 0.122 | -0.024 | 0.258 | 0.122 | -0.024 | 0.258 |  | 0.116 | -0.020 | 0.255 | 0.116 | -0.02 | 0.255 |  | 0.126 | -0.03 | 0.265 |
| NA ON | NA&1 | **0.428** | **0.322** | **0.526** | **0.428** | **0.322** | **0.526** |  | **0.419** | **0.316** | **0.511** | **0.419** | **0.316** | **0.511** |  | **0.446** | **0.34** | **0.536** |
| TM ON | TM&1 | -0.039 | -0.198 | 0.140 | -0.039 | -0.198 | 0.140 |  | 0.004 | -0.173 | 0.190 | 0.004 | -0.173 | 0.19 |  | -0.027 | -0.183 | 0.154 |
| CI ON | CI&1 | 0.003 | -0.124 | 0.140 | 0.003 | -0.124 | 0.140 |  | -0.009 | -0.143 | 0.132 | -0.009 | -0.143 | 0.132 |  | 0.01 | -0.122 | 0.151 |
| DI ON | DI&1 | 0.115 | -0.011 | 0.242 | 0.115 | -0.011 | 0.242 |  | 0.091 | -0.047 | 0.213 | 0.091 | -0.047 | 0.213 |  | 0.103 | -0.029 | 0.223 |
| TS ON | TS&1 | 0.077 | -0.100 | 0.211 | 0.077 | -0.110 | 0.211 |  | 0.066 | -0.111 | 0.206 | 0.066 | -0.111 | 0.206 |  | 0.064 | -0.096 | 0.194 |
|  |  |  |  |  |  |  |  |  |  |  |  |  |  |  |  |  |  |  |
| within level covariance/correlation | | |  |  |  |  |  |  |  |  |  |  |  |  |  |  |  |  |
| PA WITH | NA | **-0.016** | **-0.023** | **-0.009** | **-0.227** | **-0.315** | **-0.136** |  | **-0.016** | **-0.023** | **-0.009** | **-0.225** | **-0.313** | **-0.132** |  | **-0.016** | **-0.023** | **-0.009** |
| PA WITH | TM | **0.051** | **0.024** | **0.082** | **0.180** | **0.087** | **0.277** |  | **0.049** | **0.022** | **0.078** | **0.173** | **0.079** | **0.267** |  | **0.053** | **0.026** | **0.083** |
| PA WITH | CI | **0.058** | **0.037** | **0.081** | **0.240** | **0.158** | **0.324** |  | **0.055** | **0.034** | **0.078** | **0.234** | **0.149** | **0.316** |  | **0.056** | **0.034** | **0.078** |
| PA WITH | DI | **0.036** | **0.015** | **0.059** | **0.152** | **0.064** | **0.240** |  | **0.034** | **0.013** | **0.057** | **0.144** | **0.055** | **0.233** |  | **0.032** | **0.012** | **0.054** |
| PA WITH | TS | **0.019** | **0.008** | **0.031** | **0.146** | **0.062** | **0.232** |  | **0.017** | **0.006** | **0.029** | **0.132** | **0.043** | **0.219** |  | **0.017** | **0.006** | **0.029** |
| NA WITH | TM | -0.006 | -0.016 | 0.003 | -0.066 | -0.165 | 0.031 |  | -0.007 | -0.017 | 0.002 | -0.073 | -0.167 | 0.024 |  | -0.007 | -0.017 | 0.003 |
| NA WITH | CI | **-0.022** | **-0.030** | **-0.014** | **-0.262** | **-0.352** | **-0.173** |  | **-0.021** | **-0.029** | **-0.013** | **-0.256** | **-0.34** | **-0.167** |  | **-0.021** | **-0.03** | **-0.013** |
| NA WITH | DI | 0.001 | -0.007 | 0.008 | 0.007 | -0.090 | 0.102 |  | 0.001 | -0.007 | 0.008 | 0.009 | -0.081 | 0.102 |  | 0.001 | -0.007 | 0.009 |
| NA WITH | TS | **-0.008** | **-0.013** | **-0.004** | **-0.190** | **-0.282** | **-0.099** |  | **-0.008** | **-0.013** | **-0.004** | **-0.184** | **-0.274** | **-0.093** |  | **-0.008** | **-0.013** | **-0.004** |
| TM WITH | CI | **0.051** | **0.021** | **0.083** | **0.152** | **0.062** | **0.239** |  | **0.051** | **0.02** | **0.083** | **0.152** | **0.062** | **0.238** |  | **0.053** | **0.023** | **0.083** |
| TM WITH | DI | **0.036** | **0.006** | **0.066** | **0.106** | **0.016** | **0.194** |  | **0.036** | **0.007** | **0.066** | **0.107** | **0.022** | **0.194** |  | **0.036** | **0.008** | **0.067** |
| TM WITH | TS | **0.017** | **0.002** | **0.035** | **0.096** | **0.010** | **0.188** |  | **0.017** | **0.001** | **0.034** | **0.096** | **0.005** | **0.185** |  | **0.018** | **0.001** | **0.035** |
| CI WITH | DI | **0.047** | **0.023** | **0.071** | **0.169** | **0.085** | **0.247** |  | **0.044** | **0.021** | **0.069** | **0.16** | **0.076** | **0.242** |  | **0.044** | **0.02** | **0.068** |
| CI WITH | TS | **0.035** | **0.022** | **0.049** | **0.230** | **0.146** | **0.306** |  | **0.033** | **0.02** | **0.046** | **0.218** | **0.137** | **0.297** |  | **0.033** | **0.02** | **0.047** |
| DI WITH | TS | **0.021** | **0.008** | **0.034** | **0.135** | **0.052** | **0.215** |  | **0.018** | **0.006** | **0.032** | **0.121** | **0.038** | **0.206** |  | **0.018** | **0.005** | **0.031** |
|  |  |  |  |  |  |  |  |  |  |  |  |  |  |  |  |  |  |  |
| between level covariance/correlations | | | |  |  |  |  |  |  |  |  |  |  |  |  |  |  |  |
| PA WITH | NA | -0.027 | -0.101 | 0.028 | -0.180 | -0.502 | 0.168 |  | -0.028 | -0.098 | 0.028 | -0.185 | -0.501 | 0.167 |  | -0.029 | -0.097 | 0.026 |
| PA WITH | TM | 0.035 | -0.089 | 0.178 | 0.117 | -0.268 | 0.466 |  | 0.032 | -0.086 | 0.173 | 0.108 | -0.266 | 0.455 |  | 0.033 | -0.09 | 0.178 |
| PA WITH | CI | **0.138** | **0.021** | **0.317** | **0.413** | **0.065** | **0.667** |  | **0.131** | **0.019** | **0.303** | **0.397** | **0.062** | **0.661** |  | **0.132** | **0.017** | **0.309** |
| PA WITH | DI | **0.177** | **0.056** | **0.374** | **0.503** | **0.181** | **0.722** |  | **0.168** | **0.056** | **0.35** | **0.487** | **0.174** | **0.714** |  | **0.171** | **0.05** | **0.354** |
| PA WITH | TS | 0.049 | -0.061 | 0.186 | 0.169 | -0.187 | 0.484 |  | 0.047 | -0.057 | 0.176 | 0.16 | -0.185 | 0.466 |  | 0.05 | -0.063 | 0.179 |
| NA WITH | TM | -0.010 | -0.045 | 0.017 | -0.160 | -0.525 | 0.240 |  | -0.011 | -0.043 | 0.015 | -0.174 | -0.534 | 0.221 |  | -0.012 | -0.043 | 0.014 |
| NA WITH | CI | -0.013 | -0.050 | 0.013 | -0.190 | -0.534 | 0.185 |  | -0.013 | -0.048 | 0.014 | -0.19 | -0.522 | 0.175 |  | -0.014 | -0.047 | 0.013 |
| NA WITH | DI | -0.024 | -0.064 | 0.003 | -0.331 | -0.632 | 0.048 |  | -0.024 | -0.062 | 0.001 | -0.339 | -0.637 | 0.008 |  | -0.024 | -0.062 | 0.004 |
| NA WITH | TS | -0.010 | -0.040 | 0.013 | -0.171 | -0.488 | 0.193 |  | -0.011 | -0.038 | 0.013 | -0.176 | -0.49 | 0.193 |  | -0.011 | -0.041 | 0.012 |
| TM WITH | CI | 0.052 | -0.004 | 0.137 | 0.372 | -0.029 | 0.667 |  | 0.05 | -0.005 | 0.135 | 0.369 | -0.039 | 0.673 |  | 0.049 | -0.006 | 0.13 |
| TM WITH | DI | 0.053 | -0.004 | 0.139 | 0.365 | -0.035 | 0.664 |  | 0.051 | -0.004 | 0.135 | 0.364 | -0.029 | 0.674 |  | 0.051 | -0.006 | 0.134 |
| TM WITH | TS | **0.052** | **0.007** | **0.122** | **0.427** | **0.058** | **0.702** |  | **0.051** | **0.005** | **0.123** | **0.428** | **0.042** | **0.699** |  | **0.05** | **0.006** | **0.122** |
| CI WITH | DI | **0.080** | **0.021** | **0.181** | **0.504** | **0.149** | **0.742** |  | **0.076** | **0.019** | **0.169** | **0.487** | **0.139** | **0.734** |  | **0.078** | **0.016** | **0.167** |
| CI WITH | TS | **0.049** | **0.000** | **0.122** | 0.364 | -0.003 | 0.644 |  | 0.048 | -0.001 | 0.12 | 0.361 | -0.004 | 0.642 |  | **0.05** | **0.002** | **0.123** |
| DI WITH | TS | 0.036 | -0.019 | 0.108 | 0.255 | -0.120 | 0.576 |  | 0.034 | -0.016 | 0.104 | 0.247 | -0.118 | 0.556 |  | 0.035 | -0.019 | 0.103 |
|  |  |  |  |  |  |  |  |  |  |  |  |  |  |  |  |  |  |  |
| Trends |  |  |  |  |  |  |  |  |  |  |  |  |  |  |  |  |  |  |
| POS.ON | Time |  |  |  |  |  |  |  | **0.002** | **0** | **0.004** | **0.105** | **0.016** | **0.199** |  | **0.003** | **0.001** | **0.004** |
| NEG.ON | Time |  |  |  |  |  |  |  | 0 | -0.001 | 0 | -0.009 | -0.08 | 0.061 |  | 0 | -0.001 | 0 |
| CM.ON | Time |  |  |  |  |  |  |  | 0.001 | -0.001 | 0.003 | 0.034 | -0.057 | 0.131 |  | 0.001 | -0.001 | 0.003 |
| KA.ON | Time |  |  |  |  |  |  |  | **0.003** | **0.001** | **0.004** | **0.122** | **0.029** | **0.211** |  | **0.003** | **0.001** | **0.005** |
| DI.ON | Time |  |  |  |  |  |  |  | **0.002** | **0.001** | **0.004** | **0.112** | **0.026** | **0.198** |  | **0.003** | **0.001** | **0.004** |
| TS.ON | Time |  |  |  |  |  |  |  | **0.001** | **0** | **0.002** | **0.125** | **0.038** | **0.212** |  | **0.002** | **0.001** | **0.003** |

*Note.* Table displays unstandardised and standardised regression coefficients and the corresponding 95% credible interval. Effects whose 95% credible interval does not contain zero are highlighted in bold face. *N* = 47; *N* = 653, PA = positive affect; NA = negative affect; TM = time management; CI = clarity of instruction; DI = differentiation; TS = teacher-student relationship; TM&1 = time management at the previous day; CI&1: clarity of instruction at the previous day; DI&1: differentiation at the previous day; TS&1: teacher-student relationship at the previous day; PA&1: positive affect at the previous day; NA&1: negative affect at the previous day.

^a^ rDSEM does not provide standardized parameter estimates.

**Table 2S**

*Results from the DSEM reported in the paper and bivariate DSEMs considering only one affect variable and one instructional quality measure at a time.*

|  |  | DSEM |  |  |  |  |  |  | bivariate DSEM ^a^ | |  |  |  |  |
| --- | --- | --- | --- | --- | --- | --- | --- | --- | --- | --- | --- | --- | --- | --- |
|  |  | Unstandardised Results | | | Standardised Results | | |  | Unstandardised Results | | | Standardised Results | | |
|  |  | Est. | 2.5% | 2.5% | Est. | 2.5% | 2.5% |  | Est. | 2.5% | 2.5% | Est. | 2.5% | 2.5% |
|  |  |  | CI low | CI up |  | CI low | CI up |  |  | CI low | CI up |  | CI low | CI up |
| spillover effects | |  |  |  |  |  |  |  |  |  |  |  |  |  |
| PA ON | TM&1 | **0.117** | **0.017** | **0.205** | **0.162** | **0.024** | **0.286** |  | **0.113** | **0.014** | **0.212** | **0.155** | **0.018** | **0.289** |
| PA ON | CI&1 | -0.012 | -0.132 | 0.116 | -0.014 | -0.149 | 0.134 |  | 0.025 | -0.114 | 0.174 | 0.028 | -0.133 | 0.2 |
| PA ON | DI&1 | 0.051 | -0.066 | 0.165 | 0.058 | -0.076 | 0.192 |  | 0.067 | -0.072 | 0.21 | 0.077 | -0.081 | 0.239 |
| PA ON | TS&1 | -0.019 | -0.253 | 0.219 | -0.012 | -0.157 | 0.136 |  | 0.133 | -0.281 | 0.425 | 0.083 | -0.17 | 0.262 |
|  |  |  |  |  |  |  |  |  |  |  |  |  |  |  |
| NA ON | TM&1 | 0.003 | -0.025 | 0.048 | 0.011 | -0.096 | 0.121 |  | 0.005 | -0.03 | 0.038 | 0.018 | -0.108 | 0.143 |
| NA ON | CI&1 | **0.045** | **0.002** | **0.082** | **0.137** | **0.006** | **0.254** |  | 0.043 | -0.001 | 0.082 | 0.131 | -0.002 | 0.263 |
| NA ON | DI&1 | -0.031 | -0.066 | 0.007 | -0.095 | -0.202 | 0.022 |  | -0.033 | -0.074 | 0.01 | -0.101 | -0.222 | 0.032 |
| NA ON | TS&1 | -0.037 | -0.114 | 0.039 | -0.063 | -0.193 | 0.065 |  | -0.048 | -0.14 | 0.047 | -0.079 | -0.229 | 0.08 |
|  |  |  |  |  |  |  |  |  |  |  |  |  |  |  |
| TM ON | PA&1 | 0.155 | -0.306 | 0.367 | 0.111 | -0.225 | 0.265 |  | 0.026 | -0.205 | 0.28 | 0.02 | -0.152 | 0.207 |
| TM ON | NA&1 | **-0.530** | **-0.997** | **-0.082** | **-0.143** | **-0.264** | **-0.022** |  | **-0.507** | **-0.98** | **-0.025** | **-0.137** | **-0.265** | **-0.007** |
| CI ON | PA&1 | 0.097 | -0.050 | 0.229 | 0.085 | -0.044 | 0.201 |  | 0.134 | -0.068 | 0.326 | 0.118 | -0.06 | 0.292 |
| CI ON | NA&1 | -0.190 | -0.543 | 0.175 | -0.062 | -0.177 | 0.056 |  | -0.083 | -0.49 | 0.324 | -0.027 | -0.163 | 0.104 |
| DI ON | PA&1 | -0.015 | -0.155 | 0.157 | -0.013 | -0.134 | 0.138 |  | 0.107 | -0.065 | 0.278 | 0.095 | -0.058 | 0.239 |
| DI ON | NA&1 | **-0.350** | **-0.674** | **-0.002** | **-0.114** | **-0.219** | **-0.001** |  | -0.314 | -0.68 | 0.029 | -0.103 | -0.221 | 0.009 |
| TS ON | PA&1 | 0.056 | -0.033 | 0.136 | 0.090 | -0.055 | 0.221 |  | 0.09 | -0.019 | 0.184 | 0.147 | -0.031 | 0.298 |
| TS ON | NA&1 | -0.008 | -0.211 | 0.200 | -0.005 | -0.126 | 0.118 |  | -0.013 | -0.245 | 0.188 | -0.008 | -0.148 | 0.112 |
| carry-over effects | |  |  |  |  |  |  |  |  |  |  |  |  |  |
| PA ON | PA&1 | 0.122 | -0.024 | 0.258 | 0.122 | -0.024 | 0.258 |  | **0.278** | **0.084** | **0.426** | **0.278** | **0.084** | **0.426** |
| NA ON | NA&1 | **0.428** | **0.322** | **0.526** | **0.428** | **0.322** | **0.526** |  | **0.4** | **0.278** | **0.512** | **0.4** | **0.278** | **0.512** |
| TM ON | TM&1 | -0.039 | -0.198 | 0.140 | -0.039 | -0.198 | 0.140 |  | -0.01 | -0.162 | 0.148 | -0.01 | -0.162 | 0.148 |
| CI ON | CI&1 | 0.003 | -0.124 | 0.140 | 0.003 | -0.124 | 0.140 |  | 0.078 | -0.088 | 0.229 | 0.078 | -0.088 | 0.229 |
| DI ON | DI&1 | 0.115 | -0.011 | 0.242 | 0.115 | -0.011 | 0.242 |  | 0.13 | -0.027 | 0.272 | 0.13 | -0.027 | 0.272 |
| TS ON | TS&1 | 0.077 | -0.100 | 0.211 | 0.077 | -0.110 | 0.211 |  | 0.1 | -0.176 | 0.314 | 0.1 | -0.176 | 0.314 |
|  |  |  |  |  |  |  |  |  |  |  |  |  |  |  |
| within level covariance/correlation | | |  |  |  |  |  |  |  |  |  |  |  |  |
| PA WITH | NA | -0.016 | -0.023 | -0.009 | -0.227 | -0.315 | -0.136 |  |  |  |  |  |  |  |
| PA WITH | TM | **0.051** | **0.024** | **0.082** | **0.180** | **0.087** | **0.277** |  | **0.054** | **0.025** | **0.081** | **0.187** | **0.09** | **0.273** |
| PA WITH | CI | **0.058** | **0.037** | **0.081** | **0.240** | **0.158** | **0.324** |  | **0.055** | **0.033** | **0.078** | **0.228** | **0.14** | **0.311** |
| PA WITH | DI | **0.036** | **0.015** | **0.059** | **0.152** | **0.064** | **0.240** |  | **0.03** | **0.008** | **0.052** | **0.125** | **0.034** | **0.209** |
| PA WITH | TS | **0.019** | **0.008** | **0.031** | **0.146** | **0.062** | **0.232** |  | **0.015** | **0.003** | **0.028** | **0.118** | **0.022** | **0.207** |
| NA WITH | TM | -0.006 | -0.016 | 0.003 | -0.066 | -0.165 | 0.031 |  | -0.007 | -0.017 | 0.002 | -0.072 | -0.167 | 0.022 |
| NA WITH | CI | **-0.022** | **-0.030** | **-0.014** | **-0.262** | **-0.352** | **-0.173** |  | **-0.024** | **-0.032** | **-0.016** | **-0.279** | **-0.363** | **-0.195** |
| NA WITH | DI | 0.001 | -0.007 | 0.008 | 0.007 | -0.090 | 0.102 |  | 0.001 | -0.007 | 0.008 | 0.006 | -0.082 | 0.103 |
| NA WITH | TS | **-0.008** | **-0.013** | **-0.004** | **-0.190** | **-0.282** | **-0.099** |  | **-0.008** | **-0.012** | **-0.004** | **-0.173** | **-0.262** | **-0.085** |
| TM WITH | CI | 0.051 | 0.021 | 0.083 | 0.152 | 0.062 | 0.239 |  | - | - | - | - | - | - |
| TM WITH | DI | 0.036 | 0.006 | 0.066 | 0.106 | 0.016 | 0.194 |  | - | - | - | - | - | - |
| TM WITH | TS | 0.017 | 0.002 | 0.035 | 0.096 | 0.010 | 0.188 |  | - | - | - | - | - | - |
| CI WITH | DI | 0.047 | 0.023 | 0.071 | 0.169 | 0.085 | 0.247 |  | - | - | - | - | - | - |
| CI WITH | TS | 0.035 | 0.022 | 0.049 | 0.230 | 0.146 | 0.306 |  | - | - | - | - | - | - |
| DI WITH | TS | 0.021 | 0.008 | 0.034 | 0.135 | 0.052 | 0.215 |  | - | - | - | - | - | - |
|  |  |  |  |  |  |  |  |  |  |  |  |  |  |  |
| between level covariance/correlations | | | |  |  |  |  |  |  |  |  |  |  |  |
| PA WITH | NA | -0.027 | -0.101 | 0.028 | -0.180 | -0.502 | 0.168 |  | - | - | - | - | - | - |
| PA WITH | TM | 0.035 | -0.089 | 0.178 | 0.117 | -0.268 | 0.466 |  | 0.028 | -0.053 | 0.141 | 0.116 | -0.202 | 0.469 |
| PA WITH | CI | 0.138 | 0.021 | 0.317 | 0.413 | 0.065 | 0.667 |  | 0.106 | 0.024 | 0.213 | 0.398 | 0.101 | 0.644 |
| PA WITH | DI | 0.177 | 0.056 | 0.374 | 0.503 | 0.181 | 0.722 |  | 0.14 | 0.054 | 0.275 | 0.504 | 0.211 | 0.723 |
| PA WITH | TS | 0.049 | -0.061 | 0.186 | 0.169 | -0.187 | 0.484 |  | 0.037 | -0.035 | 0.133 | 0.157 | -0.147 | 0.448 |
| NA WITH | TM | -0.010 | -0.045 | 0.017 | -0.160 | -0.525 | 0.240 |  | -0.01 | -0.032 | 0.01 | -0.195 | -0.528 | 0.189 |
| NA WITH | CI | -0.013 | -0.050 | 0.013 | -0.190 | -0.534 | 0.185 |  | -0.011 | -0.033 | 0.007 | -0.202 | -0.49 | 0.122 |
| NA WITH | DI | -0.024 | -0.064 | 0.003 | -0.331 | -0.632 | 0.048 |  | -0.02 | -0.045 | 0 | -0.346 | -0.622 | 0.002 |
| NA WITH | TS | -0.010 | -0.040 | 0.013 | -0.171 | -0.488 | 0.193 |  | -0.008 | -0.028 | 0.007 | -0.171 | -0.475 | 0.137 |
| TM WITH | CI | 0.052 | -0.004 | 0.137 | 0.372 | -0.029 | 0.667 |  | - | - | - | - | - | - |
| TM WITH | DI | 0.053 | -0.004 | 0.139 | 0.365 | -0.035 | 0.664 |  | - | - | - | - | - | - |
| TM WITH | TS | 0.052 | 0.007 | 0.122 | 0.427 | 0.058 | 0.702 |  | - | - | - | - | - | - |
| CI WITH | DI | 0.080 | 0.021 | 0.181 | 0.504 | 0.149 | 0.742 |  | - | - | - | - | - | - |
| CI WITH | TS | 0.049 | 0.000 | 0.122 | 0.364 | -0.003 | 0.644 |  | - | - | - | - | - | - |
| DI WITH | TS | 0.036 | -0.019 | 0.108 | 0.255 | -0.120 | 0.576 |  | - | - | - | - | - | - |

*Note.* Table displays unstandardised and standardised regression coefficients and the corresponding 95% credible interval. Effects whose 95% credible interval does not contain zero are highlighted in bold face. *N* = 47; *N* = 653, PA = positive affect; NA = negative affect; TM = time management; CI = clarity of instruction; DI = differentiation; TS = teacher-student relationship; TM&1 = time management at the previous day; CI&1: clarity of instruction at the previous day; DI&1: differentiation at the previous day; TS&1: teacher-student relationship at the previous day; PA&1: positive affect at the previous day; NA&1: negative affect at the previous day.

^a^ The bivariate analyses provided some estimates multiple times (e.g., carry over effects for PA and NA are estimated in four bivariate models). For reasons of clarity, we report only one estimate. Notably, the estimates can slightly differ between models, as they are partial effects. However, the pattern of results was supported in all analyses.

**Mplus Input for the multivariate DSEM**

VARIABLE:

NAMES = TIME POS NEG

KA TS CM DI ID;

MISSING are all (-99);

USEVAR = POS NEG CM KA DI TS;

CLUSTER= ID;

lagged = POS (1) NEG (1) CM (1) KA (1) DI (1) TS (1); !(1) indicates how many time units are used to "go backward"

TINTERVAL = time2 (1); !(1) indicates that the time interval amounts 1 unit of the Time scale (i.e. days)

ANALYSIS:

TYPE = TWOLEVEL ;

ESTIMATOR = BAYES;

Biter = (5000);

FBITERATIONS = 3000;

THIN = 50;

MODEL:

%WITHIN%

!autoregressive paths

POS ON POS&1;

NEG ON NEG&1;

CM ON CM&1;

KA ON KA&1;

DI ON DI&1;

TS ON TS&1;

!within correlations = correlation between two variables

!assessed at the same day (average value of within correlations)

!equals H1 and H2

POS-TS WITH POS-TS;

!H3 spill over effects: Does instructional quality on the previous day predict emotions on the next day?

POS ON CM&1;

NEG ON CM&1;

POS ON KA&1;

NEG ON KA&1;

POS ON DI&1;

NEG ON DI&1;

POS ON TS&1;

NEG ON TS&1;

!H3 spill over effects: Do emotions on the previous day predict instructional quality on the next day?

CM ON POS&1;

CM ON NEG&1;

KA ON POS&1;

KA ON NEG&1;

DI ON POS&1;

DI ON NEG&1;

TS ON POS&1;

TS ON NEG&1;

%BETWEEN%

!correlations at individual level

POS-TS WITH POS-TS;

OUTPUT: TECH1 TECH8 STDYX;

**References**

Fang, J., Wen, Z., & Hau, K. T. (2024). Mediation analyses of intensive longitudinal data with dynamic structural equation modeling. *Structural Equation Modeling: A Multidisciplinary Journal, 31*(4), 728–741. <https://doi.org/10.1080/10705511.2023.2268293>

Hamaker, E. L., Asparouhov, T., & Muthén, B. O. (2023). Dynamic structural equation modeling as a combination of time series modeling, multilevel modeling, and structural equation modeling. In R. H. Hoyle (Ed.), *Handbook of Structural Equation Modeling* (2nd ed., pp. 576–596). Guilford.

McNeish, D., & Hamaker, E. L. (2020). A primer on two-level dynamic structural equation models for intensive longitudinal data in Mplus. *Psychological Methods, 25*(5), 610–635. <https://doi.org/10.1037/met0000250>
